# Susceptibility-Weighted Magnetic Resonance Imaging (MRI) of Microbleeds in Pediatric Concussion

**DOI:** 10.1177/08830738211002946

**Published:** 2021-05-08

**Authors:** Shane Virani, Alexander Barton, Bradley G. Goodyear, Keith Owen Yeates, Brian L. Brooks

**Affiliations:** 1 Department of Pediatrics, 70402 Faculty of Kinesiology, University of Calgary, Calgary, Alberta, Canada; 2 Department of Pediatrics, Neurosciences Program, Alberta Children’s Hospital, Calgary, Alberta, Canada; 3 Department of Radiology, 2129 University of Calgary, Calgary, Alberta, Canada; 4 Department of Pediatrics, 2129 University of Calgary, Calgary, Alberta, Canada; 5 Department of Psychiatry, 2129 University of Calgary, Calgary, Alberta, Canada; 6 Hotchkiss Brain Institute, Calgary, Alberta, Canada; 7 Department of Clinical Neurosciences, 2129 University of Calgary, Calgary, Alberta, Canada; 8 Department of Psychology, 2129 University of Calgary, Calgary, Alberta, Canada; 9 Alberta Children’s Hospital Research Institute, Calgary, Alberta, Canada

**Keywords:** concussion, adolescence, pediatric, long-term, susceptibility-weighted imaging, microbleeds

## Abstract

**Objective:**

The long-term consequences of pediatric concussion on brain structure are poorly understood. This study aimed to evaluate the presence and clinical significance of cerebral microbleeds several years after pediatric concussion.

**Methods:**

Children and adolescents 8-19 years of age with either a history of concussion (n = 35), or orthopedic injury (n = 20) participated. Mean time since injury for the sample was 30.4 months (SD = 19.6). Participants underwent susceptibility-weighted imaging, rated their depression and postconcussion symptoms, and completed cognitive testing. Parents of participants also completed symptom ratings for their child. Hypointensities in susceptibility-weighted images indicative of cerebral microbleeds were calculated as a measure of hypointensity burden.

**Results:**

Hypointensity burden did not differ significantly between participants with a history of concussion and those with a history of orthopedic injury. Depression ratings (self and parent report), postconcussion symptom ratings (self and parent report), and cognitive performance did not significantly correlate with hypointensity burden in the concussion group.

**Conclusions:**

These findings suggest that at approximately 2.5 years postinjury, children and adolescents with prior concussion do not have a greater amount of cerebral microbleeds compared to those with orthopedic injury. Future research should use longitudinal study designs and investigate children with persistent postconcussive symptoms to gain better insight into the long-term effects of concussion on cerebral microbleeds.

Pediatric concussion is a significant public health problem that affects millions of youth each year.^1-3^ The long-term consequences of concussion on the developing brain are of growing concern,^4,5^ leading to increased efforts in the development and use of magnetic resonance imaging (MRI) techniques to better detect and characterize long-term alterations in brain structure and function resulting from pediatric concussion.^6,7^ Susceptibility-weighted imaging is a form of MRI that can detect the presence of cerebral microbleeds acutely and long after injury, based on the image contrast generated by the magnetic susceptibility (ie, the ability to distort magnetic fields) of biological tissues.^8,9^ In particular, the susceptibility of iron makes susceptibility-weighted imaging ideal for detecting cerebral microbleeds that may be present following brain injury.^10,11^ Susceptibility-weighted imaging studies acutely after concussion have yielded mixed findings.^12^ Although some studies report increased cerebral microbleeds following injury,^13-15^ others report no significant differences compared to control subjects.^16,17^ Thus far, only 2 susceptibility-weighted imaging studies have evaluated long-term outcomes following concussion in adult populations^18,19^: the first study found that, approximately 2.5 years after sustaining a concussion, 15% of individuals under the age of 45 had some form of hemorrhage or cerebral microbleed, compared with 0% of healthy controls^18^; the second study found that 19% of individuals possessed cerebral microbleeds 1 year after concussion, although no comparison was made to a control group.^19^ To date, no studies have evaluated long-term cerebral microbleeds in pediatric-only populations.

The clinical significance of cerebral microbleeds following concussion is also an important concern, as cerebral microbleeds may correlate with neurologic dysfunction.^20^ Acutely following concussion, individuals who present with cerebral microbleeds have poorer memory compared with individuals without cerebral microbleeds.^13^ A year after concussion, greater volumes of cerebral microbleeds are present in individuals who develop depression compared with those who do not.^19^ Although these studies suggest potential associations between the chronic presence of cerebral microbleeds following concussion and worse cognitive performance and depressive symptoms, the studies primarily involve adults. The presence and impact of cerebral microbleeds following pediatric concussion are unknown and may differ from adults, given the ongoing development of the brain in youth.

Historically, the detection of cerebral microbleeds has been labor intensive, requiring hours of manual identification and counting by trained radiologists.^21^ Recently, a novel automated detection method has been proposed to categorize hypointensities in susceptibility-weighted images indicative of cerebral microbleeds and has been termed *hypointensity burden*.^15^ Hypointensity burden is capable of characterizing cerebral microbleeds less than 3 mm in diameter, which are difficult to detect by visual inspection.^15^ Using this technique, hypointensity burden was found to be significantly increased in male (but not female) university hockey players at 2 weeks postconcussion compared with baseline values obtained at the beginning of the season.^15^ However, this study had a very small sample, consisting of 5 males and 6 females with concussion. Therefore, the present study aimed to use hypointensity burden to evaluate the presence and clinical significance of cerebral microbleeds several years after pediatric concussion. Specifically, we hypothesized that long after injury, (1) children and adolescents with a history of concussion will have increased hypointensity burden compared to children and adolescents with orthopedic injury and no concussion and (2) hypointensity burden will be correlated with worse cognitive performance and more postconcussive and depressive symptoms.

## Methods

### Participants and Procedure

Children and adolescents 8-19 years of age with either a history of concussion (n = 35) or orthopedic injury (n = 20) participated in the study. Participants were identified from research databases composed of children and adolescents who presented to concussion clinics, sports medicine clinics, or emergency departments in Calgary, Alberta, Canada. All data were collected between September 2014 and November 2016. Children and adolescents were included in the concussion group if they were diagnosed with a concussion by a physician at one of the recruitment sites at least 6 months prior to participation in the study. Concussion was defined at all recruitment sites as presenting with at least 1 new postconcussive symptom acutely following injury (eg, headache), a Glasgow Coma Scale score between 13 and 15, no loss of consciousness or a loss of consciousness of less than 30 minutes, and no post-traumatic amnesia or post-traumatic amnesia of less than 24 hours.^22^ Children and adolescents were included in the orthopedic injury group if they sustained a minor injury (as determined by a score of 3 or less on the Abbreviated Injury Scale)^23^ to any part of the body other than the head and had no prior history of concussion or traumatic brain injury. Participants were excluded from both groups if they had a neurologic disorder, a psychiatric disorder, substance abuse problems, or had a motor, visual, or hearing problems that would prevent testing through an initial phone screen. Any participants who had contraindications to MRI (eg, braces or other metal in the body) were also excluded. All participants completed a 2-hour protocol that involved MRI, cognitive testing, and questionnaires. Informed consent and assent were obtained, and the study protocol was approved by the University of Calgary Health Research Ethics Board (REB13-1199).

### Measures

#### Background history

Parents of participants completed a form that collected demographic information and background history. This form included: date of most recent injury, number of previously diagnosed concussions, injury details regarding loss of consciousness and post-traumatic amnesia, and diagnoses of attention-deficit hyperactivity disorder (ADHD), learning disability, mood disorder, and migraine.

#### MRI acquisition

All images were acquired on a 3-tesla (T) GE Discovery MR750w with a 32-channel head coil at the Alberta Children’s Hospital. Susceptibility-weighted imaging data were acquired using a gradient echo pulse sequence with an effective echo time (TE) of 23 ms, a repetition time (TR) of 54 ms, and a flip angle of 15 degrees. Slices were acquired in the axial plane with a field of view (FOV) of 220 × 220 mm and a voxel volume of (3.44 × 3.44 × 4) mm^3^. T1-weighted anatomical images were acquired using *GE’s BRAVO* sequence with a TE/TR of 8.2/3.1 ms, an inversion time of 600 ms, a flip angle 10 degrees, an FOV of 220 × 220 mm, and a voxel size of 0.86 × 0.86 × 0.8 mm^3^.

#### Depression rating

Symptoms of depression were measured using the Behaviour Assessment System for Children–2nd Edition (BASC-2).^24^ The BASC-2 self-report contains 12 items related to depression that are rated either as true or false, or on a 4-point scale from never to almost always. The BASC-2 parent report contains 13 items all rated on the 4-point scale from never to almost always. Raw scores from both the self and parent report were converted into age- and sex-adjusted T-scores.

#### Postconcussion symptom rating

Participants and their parents rated their postconcussive symptoms using the Post-concussion Symptom Inventory (PCSI).^25^ The Post-concussion Symptom Inventory contains 26 items that are rated on a 7-point Likert scale (from 0 = never to 6 =almost always). In addition to a total summed score, the Post-concussion Symptom Inventory has domain scores for cognitive, emotional, fatigue, and physical domains.^25^ The Post-concussion Symptom Inventory has also been recommended as a pediatric brain injury common data element by the National Institute of Neurological Disorders and Stroke (NINDS).^26^

#### Cognitive testing

Cognitive performance of all subjects was measured using CNS Vital Signs (CNSVS).^27^ The CNS Vital Signs is a computerized cognitive testing battery that is a recommended common data element by the National Institute of Health (NIH) pediatric traumatic brain injury outcomes workgroup.^26^ The present study used select subtests (Verbal Memory, Stroop Test, Shifting Attention Test, and Continuous Performance Task) to calculate domain scores for verbal memory, reaction time, and cognitive flexibility. CNS Vital Signs scores have been previously validated and shown to be lowered in adolescents acutely after concussion.^28,29^

### MRI Image Preprocessing and Analysis

#### Preprocessing

Susceptibility-weighted imaging data were processed using a combination of FSL^30^ and in-house python code. Phase images were unwrapped and filtered using GE software. Brain masks were extracted using FSL’s BET. Phase images were smoothed using a 2-mm Gaussian kernel and then subtracted from the original, unsmoothed phase images. A negative mask was created by setting phase values above zero to 1 and phase values below or equal to zero to 1 + (phase/pi). Following standard protocols, the negative mask was multiplied by itself 4 times and then multiplied with the magnitude images to create susceptibility-weighted images.^31^ Regions of interest, including the amygdala, caudate, cerebellum, hippocampus, insula, pallidum, putamen, and thalamus, were derived from the Harvard-Oxford subcortical predefined atlas.^32^ Subject T1-weighted images were nonlinearly registered to a 2-mm MNI template. The binarized regions of interest were then inverted to subject space, thresholded at 0.9 (to account for blurring during inversion) and again binarized.

#### Cluster analysis

**Figure 1.**
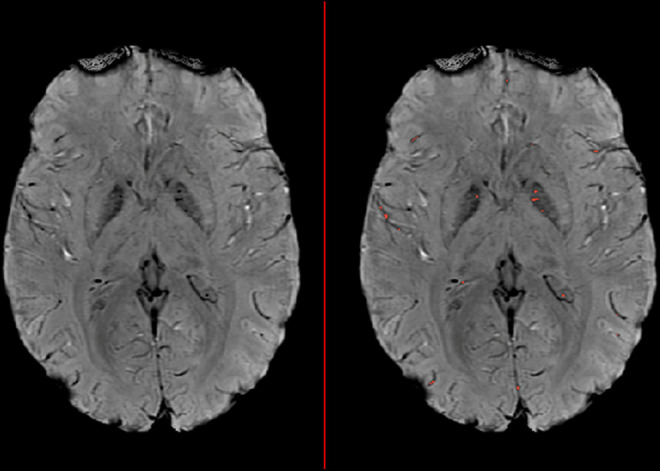
Sample susceptibility-weighted image illustrating hypointensity burden (HIB). Hypointensity clusters that pass the intensity and maximal size thresholds are highlighted in red on the right image.

Detection and clustering of micro-hypointensities proceeded according to the methods outlined by Helmer and colleagues.^15^ Maximum-intensity threshold was set to the bottom 10% of voxel intensities. After identification, these labeled clusters were passed through a Moore neighborhood clustering algorithm. Maximum cluster size was calculated to match the volume of that used in Helmer et al,^15^ resulting in a maximum cluster size of 43 voxels. No minimum cluster size was set. Hypointensity burden was calculated by summing the voxels in the final clustered image, normalizing by total brain volume and multiplying by the volume of 1 voxel. In addition to hypointensity burden, median cluster size was also extracted. An example susceptibility-weighted imaging image with hypointensity burden is shown in Figure 1.

#### Statistical analysis

Statistical analyses were carried out using a combination of SPSS 24 (IBM Corporation, Armonk, NY) and MATLAB (MathWorks Inc, Natick, MA). An analysis of covariance with sex and age as covariates was conducted to compare hypointensity burden between children and adolescents with concussions and those with orthopedic injury. Sex was taken into account in the analysis based on previous work by Helmer and colleagues that demonstrated that nonconcussed males have a higher hypointensity burden than nonconcussed females.^15^ Additionally, an exploratory analysis of covariance was conducted to investigate differences between children and adolescents who suffered 1 concussion and those who suffered multiple concussions. For other group comparisons, chi-square (χ^2^) was used for categorical data and analysis of variance was used for continuous data. All Post-concussion Symptom Inventory scores were square root transformed to correct for positive skewness. One parent did not complete the Post-concussion Symptom Inventory parent report and therefore this participant was removed from all analyses using this measure. Pearson correlations were computed to determine associations between hypointensity burden and BASC2 depression T scores (self and parent report), Post-concussion Symptom Inventory total and domain scores (self and parent report), and CNS Vital Signs domain scores. Correlations were conducted for the entire sample as well separately for participants with concussion. Independently for each set of correlations, false discovery rate (FDR) correction was used to correct for multiple comparisons.^33^

## Results

Group differences in participant characteristics, cognitive test scores, and symptom ratings are reported in Table 1. The groups were similar demographically. The concussion group consisted primarily of sport-related injuries (68%), as well as fall-related injuries (27%) and injuries from motor vehicle collisions (5%). The orthopedic injury group consisted of sport-related injuries (80%) and fall-related injuries (20%). Within the concussion group, 14 participants had 1 diagnosed concussion, 9 had 2, 9 had 3, 1 had 6, 1 had 7, and 1 had 8. The groups did not differ in terms of BASC2 depression self-report T scores, Post-concussion Symptom Inventory self-report scores, or CNS Vital Signs domain scores. Parents reported higher levels of depressive symptoms for children and adolescents with concussion than for children and adolescents with orthopedic injury, *F*(1, 52) = 7.3, *P* = .009, *η_p_* ^2^ = 0.12. Similarly, parents of participants with concussion reported higher levels of postconcussion symptoms on the Post-concussion Symptom Inventory than parents of participants with orthopedic injury, *F*(1, 52) = 10.1, *P* = .003, *η_p_* ^2^ = 0.16. Post-concussion Symptom Inventory parent-report domain scores for cognitive, emotional, and physical symptoms were all significantly higher (*P* < .05) for participants with concussion than participants with orthopedic injury.

**Table 1.** Participant Characteristics.

	Concussion (n = 35)	Orthopedic Injury Controls (n = 20)	*F* or *χ* ^2^	*P* value
Demographics				
Age, y, mean (SD)	15.0 (2.5)	14.6 (3.0)	0.38	.54
Sex (% male)	55.0	54.3	0.003	.96
Ethnicity (% Caucasian)	88.6	85.0	1.1	.59
Injury descriptors				
Time since most recent injury, mo, mean (SD)	30.5 (12.0)	30.2 (29.8)	0.002	.97
Number of concussions, median (range)	2.0 (1-8)	0	–	–
Loss of consciousness, %	34.3	0	–	–
post-traumatic amnesia, %	48.6	0	–	–
Preinjury functioning, %				
ADHD	11.8	5.3	0.60	.44
Learning disorder	11.8	0	2.4	.12
Mood/anxiety disorder	12.1	10.5	0.03	.86
Migraines	2.9	15.8	2.9	.09
Symptom ratings—self reports,^a^ mean (SD)				
BASC-2 depression *T* score	45.0 (5.1)	45.7 (6.9)	0.17	.68
PCSI total	26.6 (26.6)	15.5 (17.5)	3.5	.07
PCSI cognitive domain	6.0 (8.3)	3.9 (5.3)	1.0	.31
PCSI emotional domain	4.3 (5.2)	3.1 (4.7)	0.67	.42
PCSI fatigue domain	4.2 (3.7)	2.4 (2.7)	3.4	.07
PCSI physical domain	7.3 (8.3)	3.8 (5.8)	3.5	.07
Symptom ratings—parent reports,^a^ mean (SD)				
BASC-2 depression *T* score	53.8 (12.4)	45.7 (6.2)	7.3	.009
PCSI total	19.3 (26.6)	6.0 (9.8)	10.1	.003
PCSI cognitive domain	4.1 (6.2)	0.8 (1.8)	7.7	.008
PCSI emotional domain	4.2 (5.2)	1.7 (3.8)	5.3	.03
PCSI fatigue domain	2.5 (4.3)	0.8 (2.2)	3.2	.08
PCSI physical domain	5.1 (7.6)	1.3 (2.5)	6.9	.01
Cognitive testing, mean (SD)				
CNSVS cognitive flexibility	102.5 (16.8)	107.3 (14.4)	1.1	.29
CNSVS reaction time	99.2 (17.9)	105.2 (14.1)	1.6	.21
CNSVS verbal memory flexibility	105.2 (12.3)	99.3 (16.4)	2.3	.14

Abbreviations: ADHD, attention-deficit hyperactivity disorder; BASC-2, Behavior Assessment System for Children 2nd Edition; CNSVS, CNS Vital Signs; PCSI, Postconcussion Symptom Inventory; SD, standard deviation.

^a^ Square rooted Postconcussion Symptom Inventory scores were used for analyses.

No significant differences in whole brain hypointensity burden were found between participants with concussion and those with orthopedic injury, *F*(1, 51) = .31, *p* = .58, *η_p_* ^2^ = 0.06. Across all regions of interest, no significant differences were found between the concussion group and the orthopedic injury group. Results from all hypointensity burden comparisons between concussion and orthopedic injury participants are reported in Table 2. Additionally, no significant group differences in hypointensity burden were found in whole brain or region of interest analyses when the concussion group was divided to compare those with a single concussion to those with multiple concussions. Full results of this exploratory analysis are presented in Table 3.

**Table 2.** Hypointensity Burden Group Comparisons: Concussion Versus Orthopedic Injury Controls.

Region	Concussion,	Orthopedic injury controls,	*F* or *χ* ^2^	*P* value	*η_p_* ^2^
	mean (SD) (n = 35)	mean (SD) (n = 20)			
Whole brain	1.6 × 10^–4^ (5.0 × 10^–5^)	1.6 × 10^–4^ (2.5 × 10^–5^)	.31	.58	.006
Amygdala	6.7 × 10^–5^ (7.7 × 10^–5^)	6.7 × 10^–5^ (9.6 × 10^–5^)	.005	.95	.000
Caudate	1.8 × 10^–4^ (1.1 × 10^–4^)	2.4 × 10^–4^ (1.7 × 10^–4^)	2.5	.12	.05
Cerebellum	1.0 × 10^–4^ (3.5 × 10^–5^)	9.7 × 10^–5^ (4.0 × 10^–5^)	.17	.68	.003
Hippocampus	1.3 × 10^–4^ (1.3 × 10^–4^)	1.6 × 10^–4^ (1.5 × 10^–4^)	.47	.50	.009
Insula	1.9 × 10^–4^ (9.9 × 10^–5^)	1.8 × 10^–4^ (9.1 × 10^–5^)	.65	.42	.01
Pallidum	8.5 × 10^–4^ (7.1 × 10^–4^)	8.3 × 10^–4^ (6.3 × 10^–4^)	.03	.87	.001
Putamen	7.8 × 10^–5^ (8.4 × 10^–5^)	1.1 × 10^–4^ (9.3 × 10^–5^)	1.9	.17	.04
Thalamus	1.1 × 10^–4^ (7.5 × 10^–5^)	1.0 × 10^–4^ (5.8 × 10^–5^)	.07	.79	.001

Abbreviation: SD, standard deviation.

**Table 3.** Hypointensity Burden Group Comparisons: Single Concussion Versus Multiple Concussion.

Region	Single concussion,	Multiple concussions,	*F* or *χ* ^2^	*P* value	*η_p_* ^2^
	mean (SD) (n = 14)	mean (SD) (n = 21)			
Whole brain	1.6 × 10^–4^ (3.2 × 10^–5^)	1.5 × 10^–4^ (1.8 × 10^–5^)	.88	.35	.03
Amygdala	5.7 × 10^–5^ (5.5 × 10^–5^)	7.3 × 10^–5^ (9.0 × 10^–5^)	.40	.53	.01
Caudate	1.7 × 10^–4^ (1.3 × 10^–4^)	1.8 × 10^–4^ (1.0 × 10^–4^)	.01	.92	.000
Cerebellum	1.1 × 10^–4^ (4.5 × 10^–5^)	9.4 × 10^–5^ (2.6 × 10^–5^)	1.4	.24	.04
Hippocampus	1.6 × 10^–4^ (1.4 × 10^–4^)	1.2 × 10^–4^ (1.2 × 10^–4^)	1.3	.26	.04
Insula	1.9 × 10^–4^ (1.1 × 10^–4^)	2.0 × 10^–4^ (9.2 × 10^–5^)	.38	.54	.01
Pallidum	6.4 × 10^–4^ (6.1 × 10^–4^)	9.8 × 10^–4^ (7.6 × 10^–4^)	2.3	.14	.07
Putamen	9.8 × 10^–5^ (1.0 × 10^–4^)	6.5 × 10^–5^ (6.7 × 10^–5^)	1.5	.22	.05
Thalamus	1.2 × 10^–4^ (6.4 × 10^–5^)	9.8 × 10^–5^ (8.1 × 10^–5^)	1.4	.25	.04

Abbreviation: SD, standard deviation.

After FDR correction, a significant positive correlation was found between amygdala hypointensity burden and parent report Post-concussion Symptom Inventory emotional domain scores in the entire sample (*r* = .36, *P* = .007). However, this correlation was not significant in the concussion group only (*r* = .15, *P* = .39). No other significant correlations with hypointensity burden were found for BASC-2 depression scores (self or parent report), Post-concussion Symptom Inventory scores (self or parent report), or CNS Vital Signs domain scores. This was true when examining correlations for the entire sample as well as within the concussion group separately.

## Discussion

The present study is the first to investigate the presence of cerebral microbleeds years after pediatric concussion in order to improve our understanding of the long-term impact of these injuries. The results provide evidence that, at approximately 2.5 years postinjury, children and adolescents who sustain a concussion do not have an increased hypointensity burden compared to children and adolescents who sustain an orthopedic injury. Additionally, individuals who sustain multiple concussions do not have an increased hypointensity burden compared to individuals who have had only one concussion. These findings contrast with those of Trifan et al,^18^ who examined cerebral microbleeds 2.5 years postconcussion in a predominantly adult sample and found that individuals with a history of concussion had a greater incidence of cerebral microbleeds than a healthy control group. The prevalence of cerebral microbleeds rises with increased age^34^ and the number of cerebral microbleeds can decrease over time in individuals with chronic traumatic brain injury.^35^ Considering that our pediatric sample was on average 2.5 years postconcussion, any acute hemosiderin deposits in the brains of our participants may have had ample time to undergo evolution^35^ and dissipation. However, an overall increase in cerebral microbleeds may not have been present acutely after concussion in the sample, as studies investigating pediatric concussion and cerebral microbleeds at acute time points have been sparse and have yielded mixed findings.^14,17^ Additionally, a diffusion tensor imaging^36^ and a cortical morphometry study^37^ conducted in the same sample as the present study both found no group differences between youth with a history of concussion and those with a history of orthopedic injury years after injury. Taking all studies into account, no overall structural abnormalities are apparent for this sample approximately 2.5 years after pediatric concussion. In contrast, other functional abnormalities, such as alterations in cerebral blood flow,^38^ functional connectivity,^39^ and *N*-acetyl-aspartate,^40^ were still apparent at this time point in the same sample. Taken together, these results suggest the possibility of persistent functional but not structural alterations in the brain years after concussion.

The methods of the present study aimed to use the techniques of Helmer and colleagues to more precisely detect cerebral microbleeds through a measure of hypointensity burden.^15^ To our knowledge, their study is the only to date that has longitudinally tracked hypointensity burden following concussion. They reported that a small number of male university hockey players presented with increased hypointensity burden at 2 weeks postconcussion compared to baseline values obtained at the beginning of the hockey season. However, this finding was not replicated in a similarly small number of concussed female hockey players. Additionally, no significant changes in hypointensity burden compared to baseline were evident at other follow-up time points, including 2 months postconcussion and the end of the hockey season, suggesting normalization of hypointensity burden over time. However, these findings are severely limited because of the small sample of the study. Although the concussion group initially consisted of 5 male and 6 female patients, only 2 male and 3 female patients completed both baseline and end of season imaging. In the future, longitudinal studies with larger samples are needed to more accurately investigate the progression of cerebral microbleeds and hypointensity burden following pediatric concussion.

We also found that hypointensity burden was not correlated with symptom severity in the concussion group. Helmer and colleagues also found no correlation between hypointensity burden and Sport Concussion Assessment Tool-2 (SCAT-2) symptom scores.^15^ A previous study by Wang et al^19^ showed that individuals who develop depression following a concussion have a higher incidence of cerebral microbleeds than individuals who do not develop depression following concussion. In the present study, one concussion participant and no orthopedic injury participants (based on self-reports) and 6 concussion participants and no orthopedic injury participants (based on parent-reports) were classified as having clinically significant depression according to BASC-2 guidelines of having a *t* score of 70 or greater.^24^ Thus, the majority of our sample fall within the normal range of depressive symptoms, so we cannot compare those with clinical depression to those without. The Wang et al^19^ study unfortunately did not conduct comparisons between their nondepressed concussion participants and a control group. In a similar vein, Trifan et al,^18^ who found a greater incidence of cerebral microbleeds in concussion versus control participants, only included persistently symptomatic concussion participants who were involved in litigation and asymptomatic controls without concussion. In contrast, our study included pediatric participants with a history of concussion regardless of whether they were persistently symptomatic or not. Therefore, our sample is likely less symptomatic than the samples in Trifan et al^18^ and Wang et al,^19^ and this difference may potentially explain why we did not find a significant association between hypointensity burden and symptoms.

Cognitive test scores also were not associated with hypointensity burden. Previous research in pediatric^14^ and adult^13^ concussion samples at more acute time points have demonstrated that individuals with CMBs have worse processing speed and digit span scores. However, Helmer and colleagues reported no correlations between hypointensity burden and Immediate Post concussion Assessment and Cognitive Test (ImPACT) scores. The lack of an association in the present study suggests that the presence of hemosiderin deposits are not associated with decreased cognitive performance long term following concussion, although an association could still exist at earlier time points.

The current study has some limitations that should be noted. As mentioned previously, we do not know whether hypointensity burden increased acutely following concussion because of the cross-sectional study design. Future longitudinal studies are needed to address this limitation as well as to provide acute symptom and imaging data, which were unavailable for this study. Additionally, the current study could be subject to selection bias (ie, the participants were restricted to only youth presenting to a clinic or hospital following injury in Alberta, Canada). Because of this, and the homogeneity of the demographics of this population, the generalizability of this study may be limited and may not be representative of the broader pediatric concussion population. Third, background history for this study was obtained via parent report, which may be subject to recall bias. Parents only reported previous concussions that were diagnosed by a clinician and thus some participants may have sustained more concussions than reported in this study if they were undiagnosed. Additionally, the selection of an optimal control group for concussion studies has been debated.^41,42^ The present study used an orthopedic injury control group, which has been proposed to have similarities to concussion groups with respect to premorbid characteristics.^41^ However, recent evidence has emerged showing differences in other structural imaging modalities can be absent between concussion and orthopedic injury controls in the acute and sub-acute recovery periods, yet present between concussion and nontraumatic typically developing controls.^41^ Thus, in the present study, a comparison to typically developing controls would have been valuable in helping determine whether hypointensity burden for both the concussion and orthopedic injury groups differ from those with no history of trauma. Lastly, the modest sample size (n = 55) may have limited our ability to detect significant differences between groups. This is especially true for the correlation analyses given the low number of participants who were highly symptomatic. However, other studies using susceptibility-weighted imaging in concussion samples have had similar if not fewer participants,^13,15-17^ and studies in pediatric populations are scarce.^14,17^ Therefore, the current sample size is reasonable within the context of existing research.

In summary, this is the first published study to examine HBI in a pediatric population more than 2 years postconcussion. The findings provide evidence that HBI is not significantly increased long after pediatric concussion and that increases in hypointensity burden are not associated with long-term postconcussion symptoms or worse cognitive performance. Future research should use longitudinal study designs and investigate children with persistent postconcussive symptoms to gain better insight into the long-term effects of concussion.
